# What’s New on Quantitative CT Analysis as a Tool to Predict Growth in Persistent Pulmonary Subsolid Nodules? A Literature Review

**DOI:** 10.3390/diagnostics10020055

**Published:** 2020-01-21

**Authors:** Andrea Borghesi, Silvia Michelini, Salvatore Golemi, Alessandra Scrimieri, Roberto Maroldi

**Affiliations:** 1 Department of Radiology, University and ASST Spedali Civili of Brescia, Piazzale Spedali Civili 1, 25123 Brescia, Italy; golemisalvatore@gmail.com (S.G.); ascrimieri@alice.it (A.S.); roberto.maroldi@unibs.it (R.M.); 2 Department of Radiology, Fondazione Poliambulanza Istituto Ospedaliero, Via Leonida Bissolati, 57, 25124 Brescia, Italy; silviamichelini2015@gmail.com

**Keywords:** pulmonary nodule, subsolid nodule, ground-glass nodule, non-solid nodule, part-solid nodule, multidetector computed tomography, computer-assisted image analysis

## Abstract

Pulmonary subsolid nodules (SSNs) are observed not infrequently on thin-section chest computed tomography (CT) images. SSNs persisting after a follow-up period of three to six months have a high likelihood of being pre-malignant or malignant lesions. Malignant SSNs usually represent the histologic spectrum of pulmonary adenocarcinomas, and pulmonary adenocarcinomas presenting as SSNs exhibit quite heterogeneous behavior. In fact, while most lesions show an indolent course and may grow very slowly or remain stable for many years, others may exhibit significant growth in a relatively short time. Therefore, it is not yet clear which persistent SSNs should be surgically removed and for how many years stable SSNs should be monitored. In order to solve these two open issues, the use of quantitative analysis has been proposed to define the “tailored” management of persistent SSNs. The main purpose of this review was to summarize recent results about quantitative CT analysis as a diagnostic tool for predicting the behavior of persistent SSNs. Thus, a literature search was conducted in PubMed/MEDLINE, Scopus, and Web of Science databases to find original articles published from January 2014 to October 2019. The results of the selected studies are presented and compared in a narrative way.

## 1. Introduction

On thin-section chest computed tomography (CT) images, a subsolid nodule (SSN) is a nuanced nodular opacity that does not completely erase the underlying bronco-vascular structures of the lung [1]. SSNs, also called ground-glass nodules, are observed not infrequently in clinical practice and lung cancer screening programs [2,3,4,5], and their incidence is constantly increasing, particularly with the technological improvement of multidetector CT scanners and the broad availability of computer-aided detection software [5,6,7].

**Figure 1:**
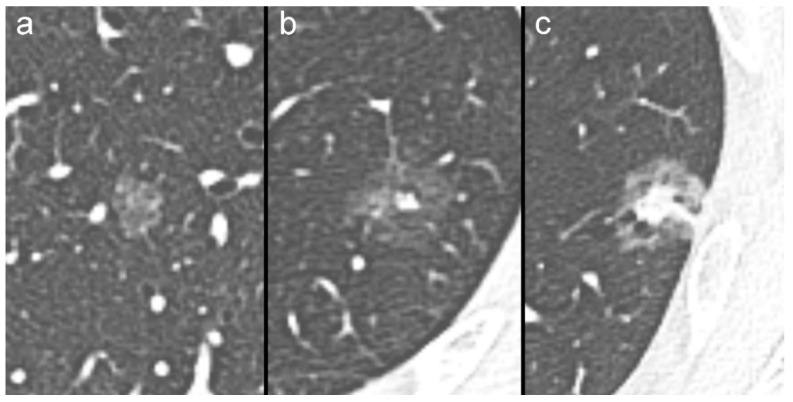
Cropped axial computed tomography (CT) images showing the different subtypes of pulmonary subsolid nodules: (**a**) non-solid nodule; (**b**) part-solid nodule with small solid component (less than 6 mm in diameter); (**c**) part-solid nodule with a large solid component.

SSNs can be the expression of both benign and malignant lesions, such as inflammation, organizing pneumonia/focal interstitial fibrosis, or pre-invasive and invasive neoplasms [8,9,10]. SSNs are conventionally divided into non-solid (NSNs) and part-solid nodules (PSNs) according to the absence or presence of an intralesional solid component (Figure 1) [11,12]. PSNs can be further classified into two or three different groups according to the size of the intralesional solid component (Figure 1) [11,12,13].

In these different groups of SSNs, the risk of aggressive behavior is strongly associated with the presence and size of the intralesional solid component [11,12,13,14]. Therefore, the updated Fleischner Society and Lung CT Screening Reporting and Data System (Lung-RADS) guidelines recommend different management for these different SSN subtypes [11,13].

Regardless of the route of presentation (clinical practice or lung cancer screening), SSNs with a solid component less than 6 mm in diameter (Figure 1a,b) are considered to have a very low or low probability of becoming aggressive cancer [13]. Therefore, conservative management with CT surveillance is justified for this subtype of SSN [11,12,13,14].

On the other hand, SSNs with a solid component greater than 6 or 8 mm (Figure 1c) are considered suspicious or very suspicious, with a high probability to be or to become aggressive cancers. In this last scenario, a more aggressive approach with additional imaging tests (such as CT at three to six months and/or PET/CT), nonsurgical tissue sampling, and/or surgical resection should be recommended, particularly for SSNs with suspicious morphology, such as irregular or spiculated margins and intralesional bubbly lucencies [11,12,13].

SSNs that persist after a follow-up period of three to six months have a high likelihood of being pre-malignant or malignant lesions [8,9,10,11,12,13,14,15]. Malignant SSNs usually represent the histologic spectrum of pulmonary adenocarcinoma with lepidic growth pattern, including pre-invasive (adenocarcinoma in situ) and invasive lesions (minimally invasive and lepidic-predominant adenocarcinoma) [16]. Very rarely, malignant SSNs may be the manifestation of primitive pulmonary lymphomas [17] and pulmonary metastases from extrapulmonary malignancies [18,19].

Pulmonary adenocarcinomas presenting as SSNs exhibit quite heterogeneous behavior [20]. While most lesions show an indolent course and may grow very slowly or remain stable for many years (especially NSNs), others (particularly PSNs with a solid component ≥ 8 mm) may exhibit a significant growth in size and/or density in a relatively short period of time (few years) [8,11,12,13,14,15]. However, it has been reported that some pulmonary adenocarcinomas presenting as NSNs can grow faster than PSNs, while some pulmonary adenocarcinoma presenting as PSNs can remain unchanged for a long period of time, similarly to NSNs [14,20]. Moreover, it is well known that not all adenocarcinomas presenting as SSNs will become clinically evident cancer in the course of life [8,11,12,13,14,15].

From a clinical point of view, the two main open questions about persistent SSNs still awaiting full resolution are: What SSNs should be surgically resected? How long should a stable or slow-growing SSN be followed?

With regard to these questions, the most fascinating goal of the radiological evaluation is undoubtedly to extract from radiological images quantitative features that may help in the early discrimination between aggressive and indolent SSNs.

Among the radiological diagnostic methods applied in the thoracic field, CT is the most widely used for quantitative analysis [21]. While the effectiveness of quantitative CT applications for predicting malignancy in solid nodules is well established and widely described in the literature [22,23], those related to SSN growth prediction are much more recent and, therefore, less well-known.

Therefore, the main purpose of this review is to summarize in a narrative way the recently emerged novelties about quantitative CT analysis as a diagnostic tool for predicting the behavior of persistent SSNs.

## 2. Materials and Methods

### 2.1. Literature Search

A literature search on three main databases (PubMed, Scopus, and Web of Science) was conducted to find relevant articles about the role of quantitative CT analysis in predicting the growth of persistent SSNs. Different combinations of the following keywords were used for this search: (a) subsolid nodule; (b) non-solid nodule; (c) part-solid nodule; (d) ground-glass nodule; (e) ground-glass opacity; (f) computed tomography; (g) quantitative analysis; (h) computer-assisted; (i) computer-aided; (j) growth; and (k) behavior. The literature search was completed on October 31st, 2019.

### 2.2. Selection Criteria

Only articles in English published from January 2014 to October 2019 that assessed the role of quantitative/computer-assisted CT analysis as a tool to predict the growth/behavior of persistent SSNs were selected and retrieved for this review.

Articles that only used nodule size as a quantitative parameter to predict nodule growth and those without a clear distinction between NSNs and PSNs were excluded. Articles that evaluated the role of quantitative CT analysis only for predicting histological invasiveness of SSNs were also excluded.

In addition, the following types of articles were also excluded from the selection: (a) review articles; (b) case reports/case series; (c) editorials/commentaries; and (d) letters. In order to permit the selection of the largest number of related articles, the references and citations of the retrieved articles were analyzed to find additional relevant studies.

All selected articles were reviewed and analyzed by two radiologists with different levels of experience in chest CT imaging and quantitative analysis (A.B and S.M. who had 15 and 8 years of experience, respectively).

### 2.3. Data Extraction

For each selected article, the following data were collected: (a) study details (first author, year, design); (b) patient characteristics (number, age, gender, smoking habits, oncologic history, country of origin); (c) SSN characteristics (number, subtype, size, location); (d) CT technical parameters (radiation dose, slice thickness); (e) quantitative CT analysis (software, segmentation modality, definition of growth); (f) quantitative CT features/parameters used as predictors of growth/behavior. The results of each study are presented in chronological order and in a narrative manner.

## 3. Results

According to the literature search and selection criteria, only seven original articles met the inclusion criteria and were considered for this review. The main characteristics of the included studies are listed in Table 1 and Table 2.

In 2014, Tamura et al. [24] and Eguchi et al. [25] published two independent retrospective studies including a total of 177 patients with single or multiple NSNs. Both studies tested some clinical and quantitative features, such as smoking history, lung cancer history, number of NSNs per patient, NSN diameter, and mean CT attenuation (m-CTA) [24,25]. In both studies, nodule segmentation was performed manually only on the cross-sectional image containing the largest nodule diameter. With regard to the definition of growth, both studies defined nodule growth as an increase in the nodule diameter by ≥2 mm or the emergence of an intralesional solid component [24,25].

In multivariable analysis, these studies found that m-CTA was an independent predictive factor for nodule growth (*p* ≤ 0.002) [24,25]. In both studies, the maximum sensitivity and specificity for predicting the growth in NSNs were obtained using a similar density cutoff value (m-CTA of about −670 UH). However, while in the paper of Eguchi et al. [25] the values of sensitivity (SE) and specificity (SP) at this cutoff value and the area under the curve (AUC) were reported (SE, 78.1%; SP, 80.0%; AUC, 0.81), these data were not available from the paper of Tamura et al. [24].

Among the clinical features, Tamura et al. [24] found that a previous history of lung cancer was the only patient characteristic strongly associated with nodule growth, whereas Eguchi et al. [25] observed that only smoking history was predictive of growth.

In another retrospective study published in 2016, Bak et al. [26] investigated some quantitative features (such as nodule diameter, volume, mass, and density) in a group of 54 histologically confirmed NSNs (six adenocarcinomas in situ, 16 minimally invasive adenocarcinomas, and 32 invasive adenocarcinomas). Regarding the computerized analysis, the segmentation process was performed manually on cross-sectional images containing the entire nodule; nodule growth, similarly to previous studies, was defined as an increase in diameter by ≥2 mm or the emergence of an internal solid component. In their three-dimensional analysis, the authors found that NSNs with a higher 97.5th percentile CTA value and steeper slope of the increase of CTA values from the 2.5th to the 97.5th percentile exhibited a propensity for higher growth rate and the development of an intralesional solid component. They also found that the combination of these two predictive factors provided an AUC of 0.78. In this study, no statistical comparison was made between patient characteristics or the number of NSNs per patient and nodule growth.

The other four original articles were published in 2019 [27,28,29,30]. Two articles, published by Shi et al. [28] and Qi et al. [30], focused exclusively on NSNs. Among the remaining two articles, while the study of Sun et al. [27] included both NSNs and PSNs, the one by Borghesi et al. [29] included only PSNs with a solid component less than 6 mm and diameter between 6 and 15 mm.

Shi et al. [28] included 59 patients with 101 NSNs. In their sample, the authors assessed several quantitative features (volumetric and histogram parameters) in order to identify the factors predictive of nodule growth. Nodule segmentation was performed in a semiautomatic mode using three-dimensional (3D) open-source software including the entire nodule. In this quantitative analysis, the authors defined growth as an increase in NSN diameter and volume/mass by at least 2 mm and 30%, respectively.

Based on multivariable analysis, Shi et al. [28] found that only 3D maximum diameter and CTA standard deviation were independent predictors of nodule growth (*p* = 0.001). In particular, the optimal cutoff values were 10.2 mm (for 3D maximum diameter) and 50 HU (for CTA standard deviation), and the AUC values were 0.896 and 0.813, respectively [28]. No correlation was observed between clinical features (such as patient age, sex, and smoking habits) or the number of NSNs per patient and nodule growth [28].

Qi et al. included 110 NSNs from 110 patients [30]. In this sample, they evaluated the natural history of persistent NSNs with deep learning-assisted nodule segmentation. For this analysis, some quantitative (nodule diameter, m-CTA, volume and mass) and non-quantitative morphological features (lobulated sign, spiculated sign, vacuole sign, air bronchogram, pleural adhesion and retraction) were considered. Nodule segmentation was performed automatically using the Dr. Wise system based on convolution neural networks [30]. The non-quantitative morphological features were assessed by two radiologists with different experience in chest CT. The authors defined the nodule growth as an increase in nodule volume ≥20% or the emergence of an intralesional solid component. In multivariable analysis, the authors found that initial diameter, volume, and mass were the main quantitative factors predicting nodule growth (*p* ≤ 0.023). Among the non-quantitative morphological features, only the lobulated sign was an independent predictor of growth. No correlation was observed between nodule growth and other characteristics, such as patient age, sex, oncologic history, number of NSNs per patient, and nodule location.

The study of Sun et al. [27] included 86 patients with 89 SSNs (42 NSNs and 47 PSNs). The authors assessed the effectiveness of four quantitative features (m-CTA, entropy, uniformity, and energy) in predicting malignancy and growth trends of SSNs detected during a low-dose CT lung cancer screening program. The nodule segmentation was performed manually using texture analysis software on the cross-sectional images containing the most representative area of the SSNs. The authors defined growth as an increase in SSN volume by at least 20% and calculated the volume and volume doubling time (DT) of the nodules using commercial CT lung analysis software. With regard to the analysis of the growth trend (performed in 61/89 SSNs), Sun et al. [27] found that only uniformity is useful in predicting growth in NSNs (*p* = 0.026), and NSNs with low uniformity exhibiting faster growth. On the other hand, no correlation was observed between PSN growth and quantitative features. In this study, no statistical comparison was made between patient characteristics and nodule growth.

Borghesi et al. [29] included 50 patients with a single PSN and assessed several quantitative features (dimensional, densitometric, and shape parameters) in order to find useful factors for predicting nodule growth. Nodule segmentation was performed manually using an open-source software package on the largest cross-sectional area of each PSN. In their quantitative analysis, the authors defined growth as an increase by more than 11.3% (i.e., more than the coefficient of repeatability of intraobserver variability) of the linear mass density (LMD), a viable two-dimensional alternative to the mass. Moreover, to determine the nodule growth rate, the authors also calculated the LMD-DT by matching the baseline with the last available follow-up CT. Based on the results obtained in their study, Borghesi et al. [29] found that both dimensional (area, perimeter, diameter, LMD) and shape features (circularity and solidity) were significantly related to nodule growth, and the strongest association was observed with circularity and solidity (*p* < 0.001). Among the clinical features (patient age, sex, smoking habits, oncologic history, emphysema status, and PSN lobe location), only oncologic history (lung or other cancers) was strongly associated with nodule growth (*p* = 0.007).

## 4. Discussion

The progressive improvement in the spatial and temporal resolution of multidetector CT scanners and the increased availability of quantitative imaging methods have led to a significant change in the way CT images are analyzed. The effects of computer-aided diagnosis software on radiologist performance are well known. Therefore, their use in the diagnosis and management of several diseases is constantly expanding.

Among quantitative CT methods, those related to thoracic imaging are the most studied [20,22,23,31,32,33,34,35,36,37,38,39,40,41,42,43]. In particular, the applications related to the classification and management of lung nodules are the most well-known and are used in both clinical practice and lung cancer screening programs [20,22,23,31,32,33,34].

While solid pulmonary nodules are the most common, subsolid nodules are the ones with the highest malignancy rate [44]. Moreover, many authors consider persistent SSNs to represent early-stage adenocarcinoma or its precursor [8,9,10,11,12,13,14,15].

The stepwise progression of a persistent SSN from a preinvasive lesion (adenomatous atypical hyperplasia and adenocarcinoma in situ) to an invasive lesion (lepidic-predominant adenocarcinoma), is quite similar to the multistep progression of colorectal cancer from a premalignant (adenomatous polyp) into a malignant lesion (carcinoma). However, while colorectal adenomas are usually removed during a colonoscopy, pulmonary preinvasive lesions presenting as SSNs require thoracic surgery (video or robotic-assisted). Moreover, the intra-operative localization of SSNs during thoracoscopic surgery is difficult without preoperative marking techniques [45,46]. Furthermore, due to the typical indolent course of SSNs and their slow growth rate, it is possible that most SSNs will never become active cancer [8,11,12,13,14,15]. Therefore, it is not yet clear which persistent SSNs should be surgically removed and for how many years stable SSNs should be monitored [8,47,48,49].

In order to solve these two-open issues, the use of quantitative analysis has been proposed to improve the risk stratification of persistent SSNs and the definition of “tailored” management [50,51,52].

The appropriate knowledge of which quantitative methods are useful for predicting the aggressiveness of persistent SSNs is crucial in this respect, as their application could affect nodule management and future guidelines.

From this point of view, this review focuses on recent innovations in quantitative CT analysis applied as a tool to predict the future growth of SSNs.

In our literature search, limited to the last few years (from January 2014 to October 2019), we found only seven original studies, all retrospective, meeting our selection criteria (Table 1 and Table 2). All studies but one were conducted on Asian patients, and only one included a European (Italian) cohort. Five papers investigated the role of quantitative analysis in NSNs [24,25,26,28,30], while one included both PSNs and NSNs [27] and the remaining paper considered only PSNs with a solid component <6 mm [29].

The results on quantitative CT analysis retrieved from the selected articles were rather heterogeneous because different quantitative features were tested and different scanners and acquisition/reconstruction protocols (different slice thickness and radiation dose) were used for CT analysis (Table 2). Considering these differences and the limited number of articles included in this review, the comparison between the different studies was performed in a narrative manner.

Among the five papers that focused only on NSNs, 2/5 (40%) of studies found that m-CTA was an independent predictive factor of nodule growth [24,25], whereas the other 3/5 (60%) of studies did not confirm this strong association [26,28,30]. Among these three studies, two found that other density-related CT features (the 97.5th percentile of CTA, the slope of the CTA values from the 2.5th to the 97.5th percentile, and the CTA standard deviation) could be useful predictors of future nodule change [26,28]. On the other hand, the remaining study found that only size-related CT features (diameter, volume, and mass) could be useful predictors of nodule growth; however, in this paper, only m-CTA was tested [29].

In agreement with these studies, even the one that focused on both NSNs and PSNs did not confirm the usefulness of m-CTA in predicting growth [27]. The authors of this study only demonstrated the usefulness of uniformity in the group of NSNs (Table 2) [27], specifically showing that the uniformity of growing NSNs was significantly lower than that of stable NSNs.

Uniformity is a texture feature providing information on the homogeneity vs heterogeneity of the nodules. Similarly, the density-related features found in the study of Bak et al. (i.e., the slope of the CTA values from the 2.5th to the 97.5th percentile) [26] and Shi et al. (i.e., the standard deviation of the CTA) reflect the intralesional heterogeneity of NSNs. Therefore, we can conclude that heterogeneity should be an effective parameter for predicting NSN growth.

To confirm the leading role of heterogeneity in predicting NSN behavior, a promising computerized method of analysis capable of an early discrimination between growing and stable NSNs was tested in a small case series [51]. This method of analysis, performed using commercial software, provides information on the nodule heterogeneity in a very clear and straightforward manner, by creating a colored 3D surface model (named *mesh plot*) of the density/grayscale values within the NSN [51]. If an NSN has high heterogeneity (i.e., greater tendency to grow), the software will create a *mesh plot* with irregular surface morphology and several peaks, similar to a mountainous area; on the contrary, for an NSN with high homogeneity (i.e., lower trend to grow), the software will create a *mesh plot* with regular surface morphology and no peaks, similar to a hilly area [51].

In the remaining paper, which focused only on PSNs with small solid component (<6 mm) in a group of 50 European (Italian) patients, the authors found that size and shape-related features (circularity and solidity) were useful in predicting growth. Circularity is a dimensionless quantitative feature that provides information about the degree to which a nodule resembles a circle [29]. Solidity is also a dimensionless quantitative feature that measures the overall concavity of a nodule [29]. Based on the results of their study, the authors concluded that PSNs with non-spherical and/or irregular shapes, especially when associated with large size, exhibited a greater tendency to grow.

Compared to the previous literature on persistent SSNs [9], the introduction of a computerized method to assess the shape of SSNs is a real innovation, as it eliminates all the limitations of subjective analysis, thus improving the risk stratification of SSNs.

Regarding clinical features, the comparison between the selected studies is even more difficult, as some data were not available. A strong correlation between clinical features and nodule growth has only been observed in 3/7 (42.9%) of studies. According to some authors [3,15], Eguchi et al. [25] observed a strong correlation with smoking history. Differently, as reported in other studies [47,53,54] Tamura et al. [24] and Borghesi et al. [29] found that an oncological history (lung or other cancers) was a significant risk factor for growth.

Our review has three main limitations. First, it was not systematic; however, the search strategy was well defined. Second, the number of selected studies was small; however, the literature search was conducted on the three main databases (PubMed/MEDLINE, Scopus and Web of Science). Third, the limited number of selected studies and the heterogeneity of their results did not allow statistical comparison.

## 5. Conclusions

In conclusion, the results of this literature review can be summarized as follows: (a) quantitative CT analysis can be a useful tool for predicting SSN growth; (b) the role of m-CTA in predicting the growth of NSNs is controversial, as it has only been observed in two studies; (c) quantitative features reflecting nodule heterogeneity can be effective in predicting NSN growth; (d) circularity and solidity are two innovative and robust quantitative shape-related features for predicting the behavior of PSNs with a solid component <6 mm; (e) oncologic and/or smoking history could be used in combination with quantitative features to improve the risk stratification of SSNs.

## Figures and Tables

**Table 1:** Study details and patient characteristics.

First Author	Year	Design	Patient Characteristics					Country
			No.	Age * (Years)	Gender (M:F)	Smoking Habit (%)	Lung Cancer History (%)	
Tamura [24]	2014	Retrospective	53	70.8 ± 9.3	23:40 ^†^	33.3 ^†^	38.1 ^†^	Japan
Eguchi [25]	2014	Retrospective	124	64.5 ± 10.4	37:87	20.2	50.8	Japan
Bak [26]	2016	Retrospective	49	58.9 ± 8.1	26:23	40.8	NA	South Korea
Sun [27]	2019	Retrospective	86	55 (41–75)	47:39	100	0	China
Shi [28]	2019	Retrospective	59	61 (40–85)	19:40	33.9	0	China
Borghesi [29]	2019	Retrospective	50	65.5 ± 10.5	24:26	70.0	20.0 ^§^	Italy
Qi [30]	2019	Retrospective	110	54.3 ± 9.7	38:72	NA	9.1 ^^^	China

* Age is presented as mean ± standard deviation or median (range); † Characteristics of the 63 nodules included in the study; ^§^ 23/50 (46%) patients had an oncologic history (20% lung and 26% other cancers); ^ 23/110 (20.9%) patients had an oncologic history (9.1% lung and 11.8% other cancers); NA, not available.

**Table 2:** Subsolid nodule characteristics, technical aspects, and quantitative features.

First Author	Subsolid Nodule Characteristics				CT Technical Parameters		Quantitative CT Feature(s) Predictive of Growth
	No.	Subtype		Size * (mm)	Slice (mm)	X-ray Dose	
		NSN	PSN				
Tamura [24]	63	63	0	11.4 ± 4.2	2.0	SD	Mean CTA
Eguchi [25]	NA °	NA °	0	7.4 ± 2.8	1.25	32 LD 92 SD	Mean CTA
Bak [26]	54	54	0	11.7 ± 5.4	2.0–2.5	SD	97.5th PCTL of CTA Slope of CTA (from 2.5th to 97.5thPCTL)
Sun [27]	89	42	47	14.3 ^§^	1.25	LD	Uniformity ^
Shi [28]	101	101	0	8.9 ± 2.6 (S) 14.3 ± 3.6 (G)	1.0	SD	3D maximum diameter Standard deviation of CTA
Borghesi [29]	50	0	50 ^†^	11 (8.3–13.2)	1.0	SD	Area, perimeter, diameter, LMD, circularity, solidity
Qi [30]	110	110	0	8.7 ± 3.2	1.0–1.25	LD or SD ^‡^	Diameter, volume, mass

NSN, nonsolid nodule; PSN, part-solid nodule; SD, standard dose; LD, low dose; CTA, CT attenuation value; PCTL, percentile; S, stable NSNs; G, growing NSNs; LMD, linear mass density; NA, not available. * Nodule size is presented as mean ± standard deviation or median (range); ^§^ Approximately derived from available data; ° The exact number of NSNs is not available (75 patients with single and 49 patients with multiple NSNs); † PSNs with a solid component <6 mm; ^ Only in NSNs. ‡ The CT protocol was heterogeneous (LD or SD, and unenhanced or enhanced CT scan).
